# Fat Transfer for Gluteal Augmentation: An Expert Video Roundtable Discussion

**DOI:** 10.1093/asjof/ojac053

**Published:** 2022-06-09

**Authors:** Jeffrey M Kenkel, Daniel Del Vecchio, Simeon Wall, Patrick Pazmino

**Affiliations:** Department of Plastic Surgery, UT Southwestern Medical Center, Dallas, TX, USA; Mass General Hospital, Boston, MA, USA; Department of Plastic Surgery, UT Southwestern Medical Center, Dallas, TX, USA; Miami, FL, USA

The authors are pleased to present an expert video roundtable discussion on fat transfer for gluteal augmentation (Video). The discussants include a panel of leading experts in aesthetic surgery, and the video roundtable was recorded live at The Aesthetic Meeting 2022 in San Diego, CA, USA. The panelists provide an overview of the current challenges, areas of research, and education being undertaken to improve the gluteal augmentation procedure, often referred to as the “Brazilian Butt Lift.” Recognizing that the past few years have placed this procedure at the forefront of plastic surgery, the panelists discuss the research that has led to a clearer understanding of how to perform the procedure, specifically where to place the cannula, which has led to improved patient outcomes and reduced mortality rates. The Aesthetic Surgery Education and Research Foundation (ASERF) supported the research that led to 3 significant articles published in *Aesthetic Surgery Journal* (*ASJ*): (1) in 2017, Report on Mortality from Gluteal Fat Grafting: Recommendations From the ASERF Task Force (viewed more than 44,000 times and the highest Altmetric score of any article published in *ASJ*, 1052);^1^ and (2) in 2020, Improvement in Brazilian Butt Lift (BBL) Safety With the Current Recommendations from ASERF, ASAPS, and ISAPS, which has been viewed more than 31,000 times.^2^ The research and procedural techniques presented in these 2 articles have contributed to the safety of the procedure and help recognize the critical challenges of gluteal augmentation. The authors discuss the most recent publication, Practice Advisory on Gluteal Fat Grafting,^3^ which is available currently online in *ASJ.*
